# Supplementary figures and images for: Automated classification of synaptic vesicles in electron tomograms of C. elegans using machine learning
Source: PLoS One. 2018 Oct 8;13(10):e0205348. doi: 10.1371/journal.pone.0205348 (PMC6175533; doi:10.1371/journal.pone.0205348)

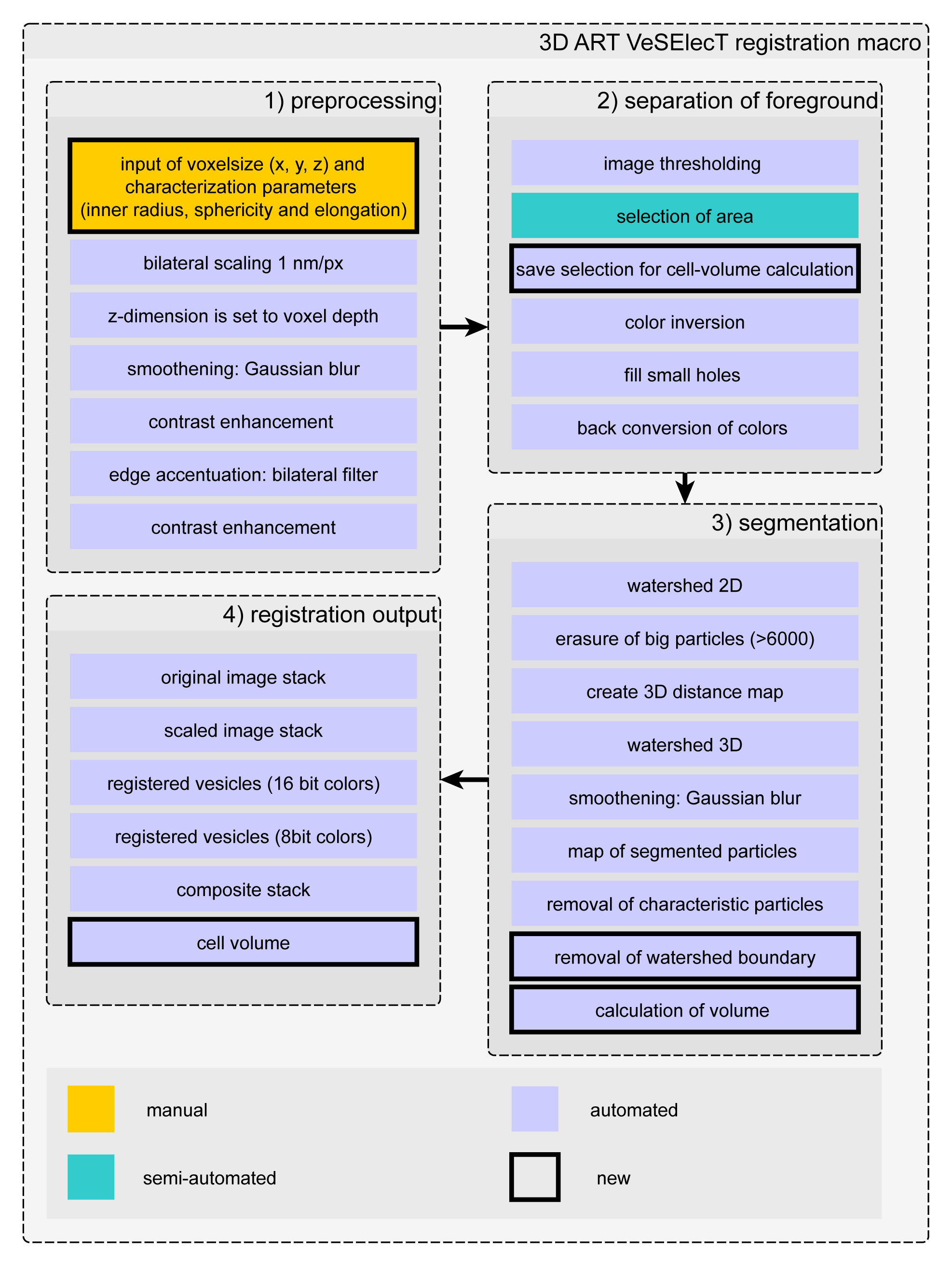

Supplement: S1 Fig — The figure shows the workflow of the previously published 3D ART VeSElecT macro [22], with changes framed in black. The macro allows the segmentation of vesicles and includes steps for the preprocessing, separation of the foreground, removal of interfering components and segmentation of individual vesicles. 1) Preprocessing: The pixel per nm resolution for the image-stack is set by user input. To allow a reproducible routine for different image resolutions and the determination of real vesicle sizes the image-stack is scaled to 1 nm/pixel in x- and y-direction using bilateral scaling. For the z-dimension, no rescaling was applied and the distance between the stack images was set to the actual voxel depth, in order to maintain the proper spatial dimensions without generating additional interpolated images. Afterwards the contrast is enhanced via histogram stretching, and the stack is then smoothed with a mean filter (radius = 3.45 px) to lower the image noise. Additionally, a 2D bilateral filter is applied for noise-reduction and edge-preservation. 2) Separation of foreground: Thresholding based on mean brightness is performed for every stack image individually. Holes within the vesicles are filled in an additional processing step. 3) Segmentation: In order to extract the round vesicles, a watershed segmentation is performed in a two-step process: first a 2D segmentation is done, then a 3D Distance map is created and smoothed with a 3D Gaussian blur of 3.45 in order to remove artifacts on the edges. Based on the created distance map, a 3D watershed segmentation is performed, which results in a map of separated particles with different colors. In a final step all particles are registered with the 3D RoiManager, and particles that don't match predefined characteristics (volume, sphericity and ellipse fitting) are automatically removed before the 4) output of the registration marks the end of the workflow. In the yellow box only one small change was made: instead of vol [file pone.0205348.s001.tif]

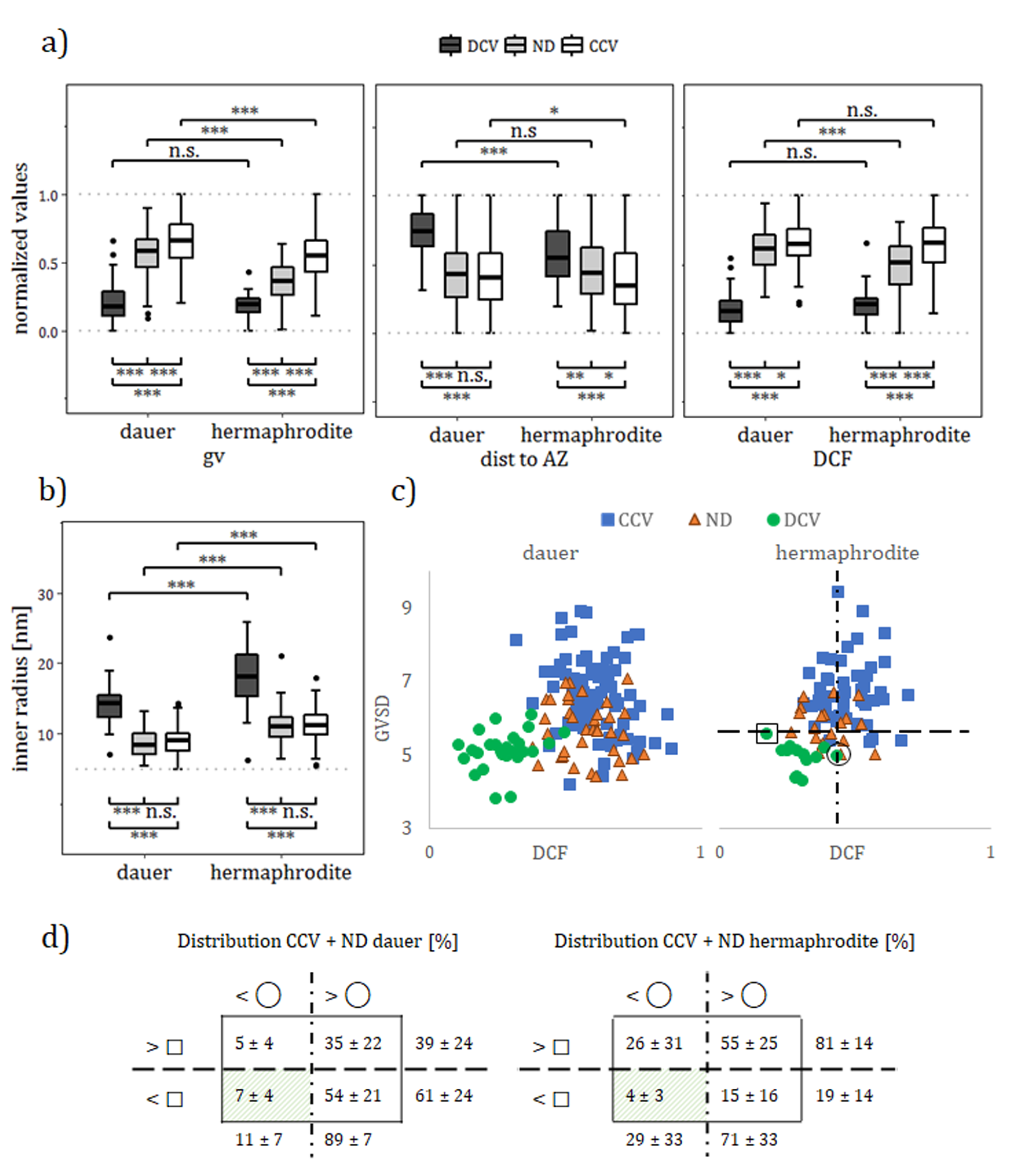

Supplement: S2 Fig — a + b) boxplots of inner radius and normalized gray value, distance to active zone and DCF of dauer larva and adult hermaphrodites. Significance levels were determined via Mann-Whitney-U: p-values > 0.05 = not significant (n.s.), < 0.05 = *, < 0.01 = **, < 0.001 = ***. c) vesicles standard deviation of gray value vs dense core factor (DCF, see S1 Text). Exemplary plots of one dauer (N2 stack01 bp) and one adult hermaphrodite (F1 s1 vnc) tomogram. Marked in hermaphrodite tomogram: DCV with highest GVSD (black square) and DCV with highest DCF (dense core factor, black circle) and corresponding quadrant thresholds (black dotted lines). d) Distribution of CCV and ND within the quadrants of all examined tomograms. The circle represents the DCV with highest DCF (= the most clear-core like DCF). The square represents the DCV with highest GVSD (= with the most clear-core like GVSD). Thresholds were independently determined for each tomogram. Green shaded quadrant (lower left) contains all DCV. (TIF) [file pone.0205348.s002.tif]

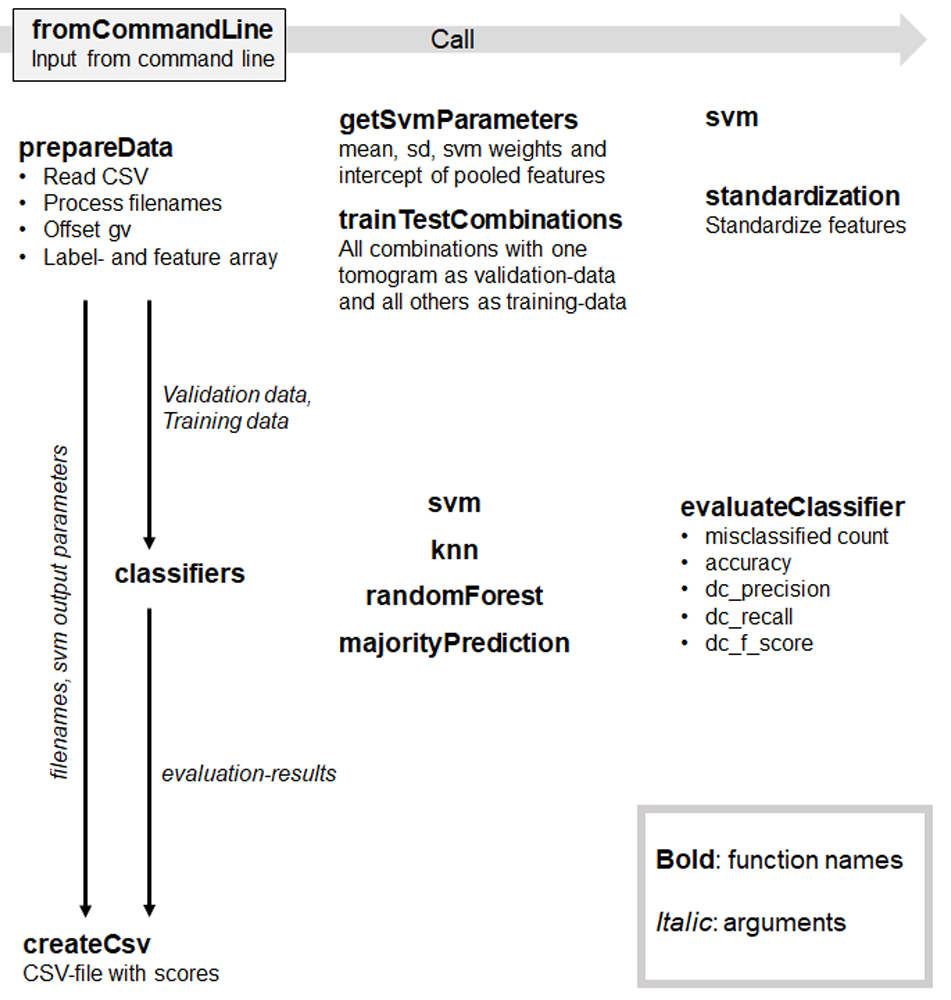

Supplement: S3 Fig — Functions are written bold, descriptions of arguments italic. Horizontal, light gray arrow indicates function calls, vertical black arrows passing of arguments. This schematic is intended to be used together with the documentation of the script (S2 Text) (TIF) [file pone.0205348.s003.tif]

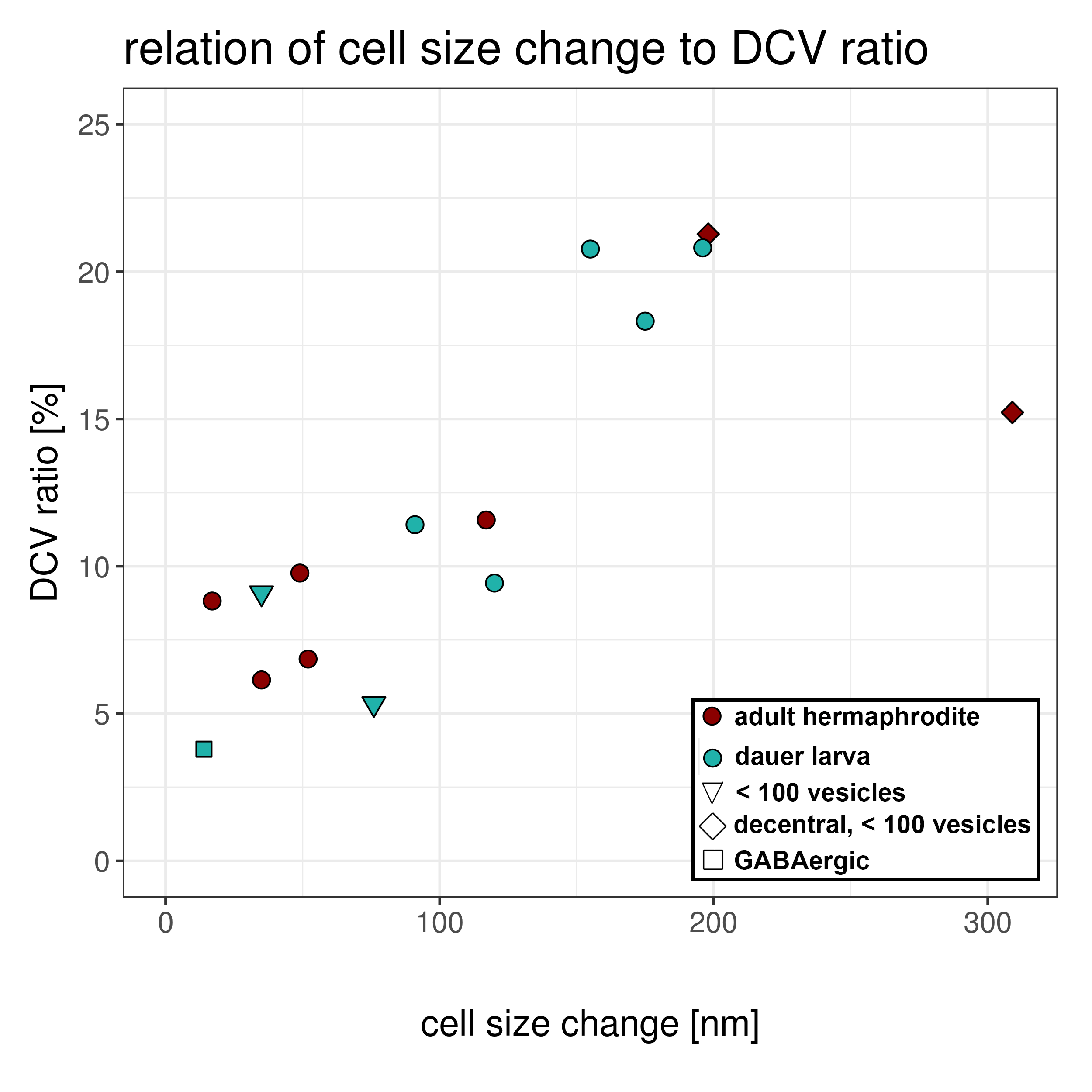

Supplement: S4 Fig — Since it is known that DCVs appear in higher concentration at the rim of the AZ [19], we compared the cell size of each neuromuscular junction (NMJ) in x and y direction in the first and last picture of each image stack. To obtain the overall cell size change, we added both differences up. This measurement gives a clue of how centrally the NMJ has been cut. For a cut near the center of the NMJ, we expect a very low cell size change rate, whereas for a more remote cut through the AZ (although they demonstrably include dense projections) we expect a higher change of the cell size since synapses tend to narrow at regions far-off the AZ. Tomograms of adult hermaphrodites are plotted in dark red. Results of dauer larvae are plotted in teal green. Tomograms of the selected set (see S1 Table, only putative cholinergic synapses with > 100 vesicles and apparent dense projections) are visualized as circles, NMJs with divergent characteristics are shown as different symbols (see legend). This shows that the number of DCVs included in a cell seems to be proportional to the cell size change. As an example, 3 of the dauer larvae tomograms show a DCV ratio of 18–21% but also have a high change of cell size. Hence, although apparent dense projections can be seen in the tomograms, the sample also includes decentral regions, were higher DCV abundance is expected. (TIF) [file pone.0205348.s004.tif]

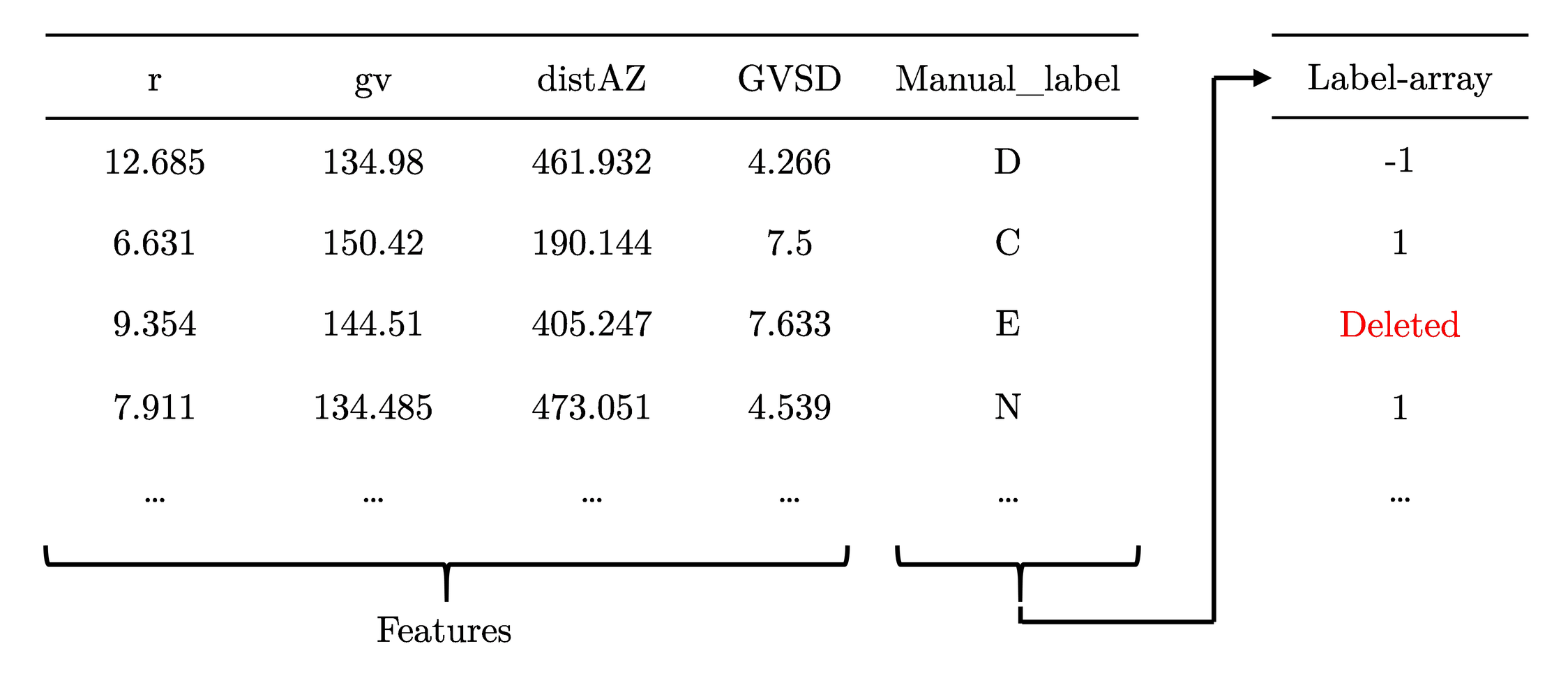

Supplement: S7 Fig — First four columns are the four features, the fifth column is the manual labels. The python script rewrites manual labels into a binary label-array: “D” (DCV) is rewritten to -1, “E” (error) is removed, and everything else is rewritten as “1” (CCV). (TIF) [file pone.0205348.s007.tif]

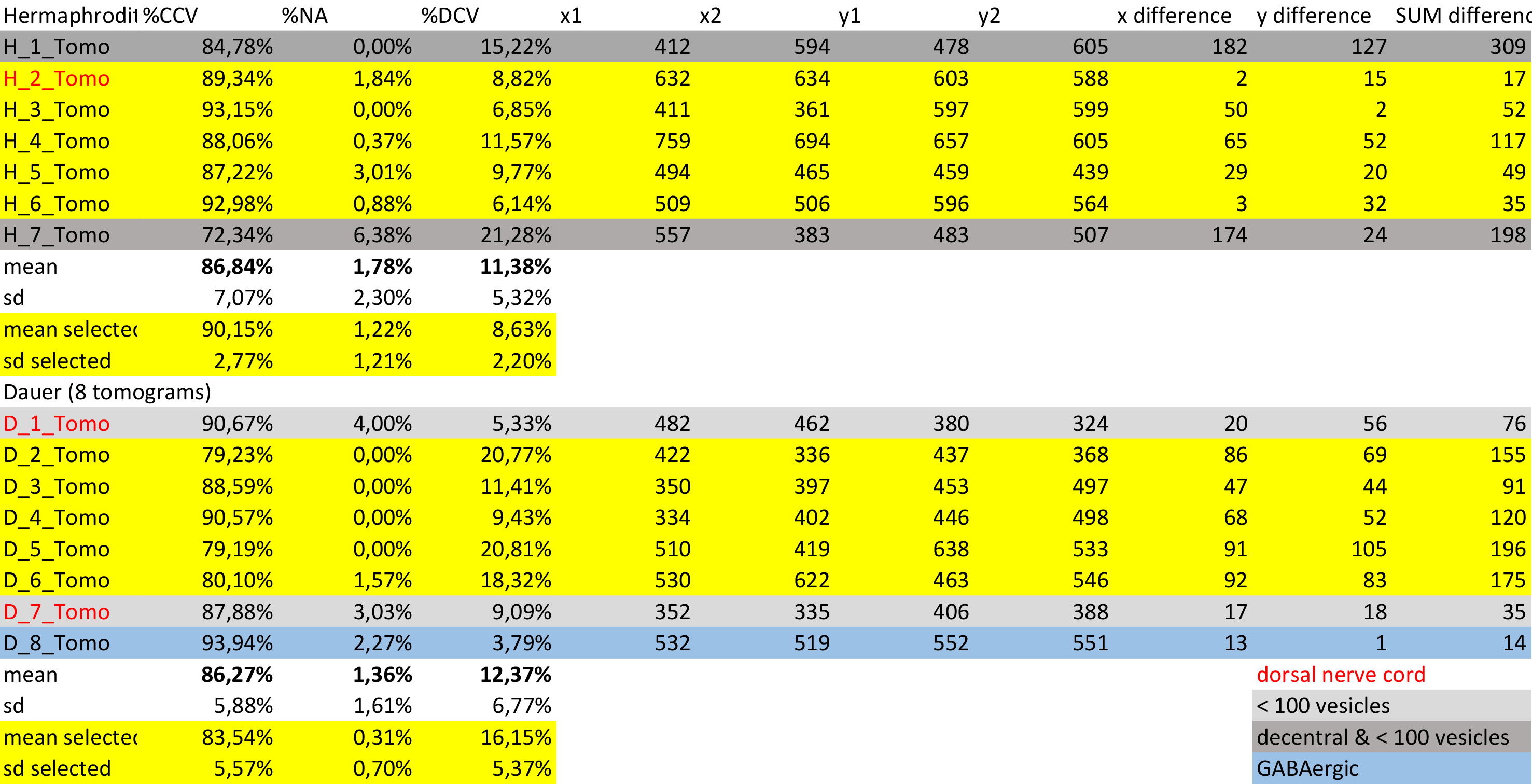

Supplement: S1 Table — All selected tomograms (> 100 vesicles, cholinergic, central cut) are marked in yellow. Other tomograms are colored after exclusion criterion in light gray (< 100 vesicles), dark gray (decentral cut plus < 100 vesicles) or blue (GABAergic NMJ). (TIF) [file pone.0205348.s008.tif]
