# Supplementary material for: Automated classification of synaptic vesicles in electron tomograms of C. elegans using machine learning
Source: PLoS One. 2018 Oct 8;13(10):e0205348. doi: 10.1371/journal.pone.0205348 (PMC6175533; doi:10.1371/journal.pone.0205348)
Supplement: S2 Table — For the random forest classifier, we use different values for the number of trees, apply the Gini criterion, no bootstrapping, and different feature numbers. For KNN, we vary the number of neighbors, the algorithm, and distance metric. For the linear SVM, we applied a one-vs-one decision function and probability estimates. For the nonlinear SVM, we tried radial basis function (RBF), polynomial and sigmoidal kernels. All other parameters were identical to those reported in the Methods section. (DOCX) [file pone.0205348.s009.docx]

| **classifier** | **accuracy** | **precision_DCV_** | **recall_DCV_** | **F-score_DCV_** |
| --- | --- | --- | --- | --- |
| **Linear SVM** | 0.98 ± 0.02 | 0.95 ± 0.07 | 0.90 ± 0.1 | 0.92 ± 0.07 |
| Linear SVM  one vs. one | 0.98 ± 0.02 | 0.95 ± 0.07 | 0.90 ± 0.1 | 0.92 ± 0.07 |
| Linear SVM  Probability estimates | 0.98 ± 0.02 | 0.95 ± 0.07 | 0.90 ± 0.1 | 0.92 ± 0.07 |
| **Random forest**  (t = 10) | 0.97 ± 0.03 | 0.88 ± 0.12 | 0.89 ± 0.09 | 0.88 ± 0.08 |
| Random forest  (t = 50) | 0.98 ± 0.02 | 0.93 ± 0.08 | 0.87 ± 0.09 | 0.90 ± 0.06 |
| Random forest  (t = 200) | 0.97 ± 0.02 | 0.92 ± 0.09 | 0.84 ± 0.14 | 0.87 ± 0.09 |
| Random forest  (t = 1500) | 0.97 ± 0.02 | 0.93 ± 0.08 | 0.86 ± 0.1 | 0.89 ± 0.07 |
| Random forest  (t = 50)  Gini criterion | 0.98 ± 0.02 | 0.93 ± 0.08 | 0.87 ± 0.1 | 0.90 ± 0.07 |
| Random forest  (t = 50)  No bootstrap | 0.97 ± 0.03 | 0.92 ± 0.11 | 0.86 ± 0.13 | 0.88 ± 0.09 |
| Random forest  (t = 50)  Max. features = 4 | 0.97 ± 0.03 | 0.91 ± 0.11 | 0.87 ± 0.1 | 0.88 ± 0.08 |
| **KNN**  (k = 10) | 0.98 ± 0.02 | 0.92 ± 0.09 | 0.89 ± 0.12 | 0.90 ± 0.09 |
| KNN  (k = 5) | 0.97 ± 0.02 | 0.94 ± 0.11 | 0.84 ± 0.14 | 0.88 ± 0.10 |
| KNN  (k = 20) | 0.98 ± 0.02 | 0.95 ± 0.08 | 0.89 ± 0.12 | 0.91 ± 0.09 |
| KNN  (k = 10)  Manhattan distance | 0.97 ± 0.02 | 0.92 ± 0.1 | 0.88 ± 0.1 | 0.89 ± 0.08 |
| KNN  (k = 10)  KD Tree Algorithm | 0.97 ± 0.02 | 0.92 ± 0.09 | 0.89 ± 0.12 | 0.90 ± 0.09 |
| **Nonlinear SVM**  RBF kernel | 0.98 ± 0.02 | 0.94 ± 0.09 | 0.89 ± 0.1 | 0.91 ± 0.08 |
| Nonlinear SVM  Polynomial kernel | 0.98 ± 0.02 | 0.95 ± 0.07 | 0.89 ± 0.1 | 0.91 ± 0.08 |
| Nonlinear SVM  Sigmoidal kernel | 0.88 ± 0.08 | 0.52 ± 0.2 | 0.51 ± 0.15 | 0.50 ± 0.15 |
