# Supplementary material for: Automated classification of synaptic vesicles in electron tomograms of C. elegans using machine learning
Source: PLoS One. 2018 Oct 8;13(10):e0205348. doi: 10.1371/journal.pone.0205348 (PMC6175533; doi:10.1371/journal.pone.0205348)
Supplement: S1 Text — Here we describe why classifying vesicles by manually thresholding a linear combination of features is not sufficient and why a machine learning approach was used instead. (DOCX) [file pone.0205348.s010.docx]

## **S1 Text:** Vesicle classification by thresholding a combination of features

## Features used for manual distinction between CCVs and DCVs were obtained using the Fiji macro 3D ART VeSElecT. S2 Fig. shows boxplots comparing gv, distAZ, radius and dense core factor (DCF) of CCVs, NDs and DCVs. The DCF is a linear weighted combination of the normalized values for distAZ, radius (r) and gv:

##

$$DCF={distAZ}_{norm}*0.25+r_{norm}*0.25+{gv}_{norm}*0.5$$

## S2a Fig. shows highly significant differences of gv between all three types of vesicles (CCVs, DCVs and NDs) for both developmental states. CCVs and NDs of dauer larvae are significantly brighter than of hermaphrodites (p-value < 0.001). Comparing dauer larvae and hermaphrodites, DCVs show no significant difference in gv. CCVs and NDs show no (dauer larvae, p-value > 0.05) or only slight differences (hermaphrodite, p-value <0.05) in their distAZ. DCVs and CCVs differ significantly (p-value < 0.01) in their distAZ in both states. DCVs and NDs show obvious differences in dauer larvae and significant differences in hermaphrodites. DCVs in dauer larvae have a significantly bigger distAZ than DCVs in hermaphrodites. Whereas CCVs and NDs in both developmental states are about the same size, DCVs show clearly higher values for radius. Young adult hermaphrodite’s vesicles are generally bigger in size compared to dauer larvae vesicles. The DCF shows that NDs and CCVs in dauer larvae differ only slightly. In young adult hermaphrodites, all vesicle types show strong differences in DCF values. Furthermore NDs differ highly significantly between developmental states, whereas CCVs and DCVs show no significant disparity.

## S2c Fig. shows results of one exemplary tomogram of dauer and young adult hermaphrodites. Here, values of GVSD are plotted against the DCF. The DCV with highest GVSD is marked with a black square, whereas DCV with highest DCF is marked with a black circle. Using these values, corresponding thresholds to separate the four quadrants are determined. All DCVs locate in the lower left quadrant. Resulting percentages of CCVs and NDs are shown in in S2d Fig. For dauer larvae, ~ 7 %, for adult hermaphrodites ~ 4 % CCVs and NDs remain in in the lower left quadrant. This shows that GVSD and DCF are important characteristics for DCV separation, but setting two thresholds is not sufficient for reliable partitioning. For that reason, we aimed at using a machine learning classifier to obtain a better separation using gv, radius, distAZ and GVSD.
