# Supplementary material for: Automated classification of synaptic vesicles in electron tomograms of C. elegans using machine learning
Source: PLoS One. 2018 Oct 8;13(10):e0205348. doi: 10.1371/journal.pone.0205348 (PMC6175533; doi:10.1371/journal.pone.0205348)
Supplement: S2 Text — This document contains detailed instructions on how to use the python script in S1 File to retrain the classifiers for your own dataset. (PDF) [file pone.0205348.s011.pdf]

How to use and adapt the Python script for training of classifiers

The main purpose of this script is to efficiently test the performance of several classifiers (k nearest neighbor (KNN), random forest, support vector machine (SVM) on hand-labeled data. Linear SVM extracted and transferred to our classification macro (see: How to use the classification macro). The script is designed to be applied to four-feature vesicle-data extracted from electron-tomograms, but can be modified to work on different input.

Software requirements

Anaconda:

Anaconda is a Python distribution for large scale data processing.

https://www.anaconda.com/download/

Python packages

- **sys**
  https://docs.python.org/3.7/library/sys.html
  http://www.open-std.org/jtc1/sc22/wg14/www/docs/n1256.pdf
- **numpy**
  http://www.numpy.org/
  Stéfan van der Walt, S. Chris Colbert and Gaël Varoquaux. **The NumPy Array: A Structure for Efficient Numerical Computation**, Computing in Science & Engineering, **13**, 22-30 (2011),
- **pandas**
  https://pandas.pydata.org/
  Wes McKinney. **Data Structures for Statistical Computing in Python**, Proceedings of the 9th Python in Science Conference, 51-56 (2010)
- **copy**
  https://docs.python.org/3/library/copy.html
- **string**
  https://docs.python.org/3/library/string.html
- **glob**
  https://docs.python.org/2/library/glob.html
- **sklearn**
  Scikit-learn: Machine Learning in Python, Pedregosa *et al.*, JMLR 12, pp. 2825-2830, 2011

conda, spyder and python version used

- python 3.5.2 and 3.6
- conda 4.4.10
- spyder 3.2.3 and 3.1.4

Format of input-data:

Point **a)** is a guide on how to format default input data. Point **b)** explains how to deal with modified input.

a) Use standard-format

Input CSV-files need to be formatted with `.` as decimal mark and `,` as delimiter. Each row should represent a measurement (= one vesicle). The first four columns should be numeric features, of which the second column must be the vesicles' gv. The fifth column contains the label. The first row should be the header. Rows labeled with a capital E (error) are deleted. Rows labeled with a capital D represent DCVs, all other labels represent CCVs. (see figure 1).

r	gv	distAZ	GVSD	Manual_label	Label-array
12.685	134.98	461.932	4.266	D	-1
6.631	150.42	190.144	7.5	C	1
9.354	144.51	405.247	7.633	E	Deleted
7.911	134.485	473.051	4.539	N	1
...	...	...	...	...	...

Features

Figure 1: Format of input-CSV

b) Modified input

All changes here are made in `def prepareData` of the script.

Different CSV-format

Search for the line `temp = pd.read_csv(filename)` in `def prepareData` (line 135 in figure 2) and change settings according to

https://pandas.pydata.org/pandas-docs/stable/generated/pandas.read_csv.html

If, for instance, you use `,` as decimal mark and `tab` as delimiter, write:

```
temp = pd.read_csv(filename, sep = '\t', decimal = ',')
```

Different features

If more than four features, or other features than the ones mentioned in **a)** should be processed, the easiest way to do so is to comment out the assertions and gv-offset (figure 2, line 137 – 145). Change column-indices (figure 2, line 147, 148, 150, marked blue) such, that the number corresponds to the number of features present.

If, for instance, the first 6 columns are features and the 7th is the label, change the number from 4 to 6.

```
134         # read in csv
135         temp = pd.read_csv(filename)
136 
137         assert np.shape(temp) == (len(temp), 5), "Data does not have 5 columns."
138         assert temp.iloc[:, 4].dtype == 'O', "Column 5 must contain labels."
139         assert all(temp.iloc[:, 4] != 'D '), "Delete whitespaces from labelvector."
140 
141         # Offset gv if darkest vesicle is brighter than 120
142         if np.min(temp.iloc[:, 1]) > 120:
143             gv_offset = np.min(temp.iloc[:, 1]) - 120
144             temp.iloc[:, 1] = temp.iloc[:, 1] - gv_offset
145             print(filename, " gv [8 bit] is offset by: ", gv_offset)
146 
147         temp = temp[temp.iloc[:, 4] != 'E']        # Exclude data with label "E" (= Error)
148         label = temp.iloc[:, 4].values             # Set column 5 as label
149         label = np.where(label == 'D', -1, 1)      # Set DCV-label as -1, others as 1
150         features = temp.iloc[:, :4].values         # Set columns 1 - 4 as features
```

comment out (yellow)

set to index of label-column (blue)

Figure 2: Positions in the script that deal with input-features are highlighted here

Running the script from an IDE (Spyder)

You can assign the paths to your csv-files in the main method at the second occurrence of `filenames`. (figure 3, line 31 -36). Please leave the first occurrence (figure 3, line 26) unchanged.

```
25 def main():
26     filenames = []  # List with filenames
27 
28 ################################################################################
29 #
30 #    # Filenames and output-csv name can be added here
31     filenames = [
32                  'example/path/to/filename1.csv',
33                  'example/path/to/anotherfile.csv',
34                  'example/path/to/theThirdFile.csv',
35                  'C:\\This\\is\\how\\To_Read_In_Files\\on_A_windows_PC.csv'
36                  ]
37     csv_name = 'classifier_results.csv'
38 #
39 ################################################################################
```

Figure 3: Position in script, where csv-files can be added

Individual file paths should be surrounded by quotation marks and separated by commas. Path separator is `/` on Linux and Mac OS and `\\` on Windows. File extension `.csv` should always be present in Windows.

You can assign the name of your output csv-file to `csv_name` using quotation marks (figure 3, line 37)

Please comment out or delete the assignment of filenames and csv_name (lines 31 – 37, if you want to run the script from the command line later.

Running the script from the command line

The script is also runnable from a unix-shell or unix-shell emulator (like Git Bash on windows), in case python is properly installed. Missing packages can be installed with

`$ conda install package-name` (when anaconda is installed).

The command is structured as indicated in figure 4.

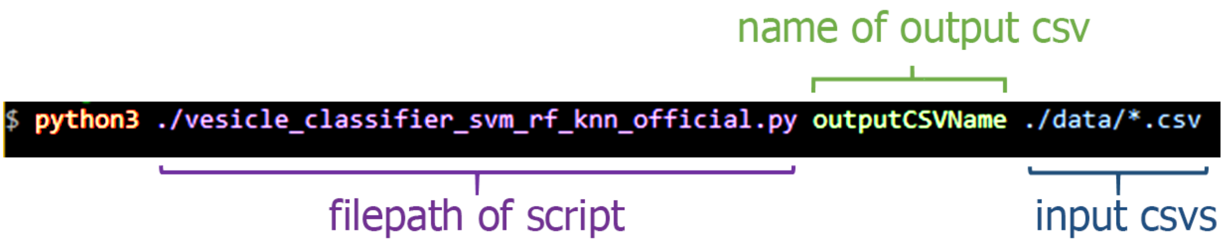

Figure 4: Command to execute script

The output csv file is saved in the current folder. The filename should not contain special characters except `.` and `_` and should not be a file path. There is no need to specify the file extension. If no csv file should be generated (i.e. for testing-purposes) `null` (without quotation-marks) can be typed instead.

If the csv-file already exists, a dialog is printed that asks whether the file should be overwritten. Affirm with `y` + enter, or type `n` + enter to enter a new filename, for which the same rules apply as mentioned above.

The **input csv** namespaces can be separated by whitespaces or collectively read in using wildcards.

Change classifier-settings

Parameters of the classifiers can be changed in the script. The classification-functions are named `svm (support vector machine)`, `knn (k nearest neighbor)` and `randomForest`. The first line of each function (figure 5, indicated by blue arrows) specifies the classifier-parameters.

```python
def svm(x_train_standard, x_test_standard, y_train, y_test):
    """Apply the svm and call the evaluation function.
    """
    svm = SVC(kernel = "linear", random_state = 0, gamma = 1, C = 1)

    svm.fit(x_train_standard, y_train)
    y_pred = svm.predict(x_test_standard)

    accuracy_list, misclassified_list, dc_precision, dc_recall, f_score = evaluateClassifier(y_test, y_pred)

    return accuracy_list, misclassified_list, dc_precision, dc_recall, f_score, y_pred


def knn(x_train_standard, x_test_standard, y_train, y_test):
    """Apply knn and call the evaluation function.
    """
    knn = KNeighborsClassifier(n_neighbors = 10, p = 2, metric = 'minkowski')

    knn.fit(x_train_standard, y_train)
    y_pred = knn.predict(x_test_standard)

    accuracy_list, misclassified_list, dc_precision, dc_recall, f_score = evaluateClassifier(y_test, y_pred)

    return accuracy_list, misclassified_list, dc_precision, dc_recall, f_score, y_pred


def randomForest(x_train_standard, x_test_standard, y_train, y_test):
    """Apply randomForest and call the evaluation function.
    """
    forest = RandomForestClassifier(criterion='entropy', n_estimators = 10, random_state = 0, n_jobs = 2)

    forest.fit(x_train_standard, y_train)
    y_pred = forest.predict(x_test_standard)

    accuracy_list, misclassified_list, dc_precision, dc_recall, f_score = evaluateClassifier(y_test, y_pred)

    return accuracy_list, misclassified_list, dc_precision, dc_recall, f_score, y_pred
```

Figure 5: classification functions

More information on possible changes can be browsed in the scikit-learn documentation:

http://scikit-learn.org/stable/documentation.html

Output

Output is a csv-file as shown in figure 6. Columns from left to right are:

A: Name of the corresponding input csv-file, acquired from shortening the filepaths

B: Accuracy

C: number of misclassified vesicles

D, E, F: precision, recall and F-score of DCV

	A	B	C	D	E	F
1	Tomogram	Accuracy	misclassified	DCV_precision	DCV_recall	DCF_F-Score
2	svm: gamma = 1, C = 1					
3	H_F2_s1_VNC_110617_features	0.95	4	0.79	0.92	0.85
4	H_M1_VNC_180717_features	0.99	1	1	0.88	0.93
5						
6	random forest:, n trees = 10, entropy					
7	H_F2_s1_VNC_110617_features	0.90	8	0.7	0.58	0.64
8	H_M1_VNC_180717_features	0.98	3	1	0.63	0.77
9						
10	knn: neighbours = 10, p = 2					
11	H_F2_s1_VNC_110617_features	0.95	4	1	0.67	0.80
12	H_M1_VNC_180717_features	0.98	2	1	0.75	0.86
13						
14	majority prediction					
15	H_F2_s1_VNC_110617_features	0.94	5	0.82	0.75	0.78
16	H_M1_VNC_180717_features	0.98	2	1	0.75	0.86
17						
18		radius	gv	dist	GVSD	intercept
19	svm_weights	-1.381	1.034	-0.576	1.332	4.405
20	mean	11.4	129.4	257.5	6.0	
21	std	2.9	3.4	102.3	1.4	

Figure 6: Output csv file

The first block of rows corresponds to the SVM, next to random forest, third to knn, fourth to a majority-prediction of the previous three.

The last block outputs SVM-parameters (red box). These can be used in the classification Macro.

Please note that the only variable fields in the output-csv are numeric fields and the file-names. Classifier-settings and order of header will not change automatically, when they are changed in the script or in the input-csv.
