# Supplementary material for: Automated classification of synaptic vesicles in electron tomograms of C. elegans using machine learning
Source: PLoS One. 2018 Oct 8;13(10):e0205348. doi: 10.1371/journal.pone.0205348 (PMC6175533; doi:10.1371/journal.pone.0205348)
Supplement: S3 Text — This document contains detailed instructions on how to apply the vesicle classification macro in S2 File. (PDF) [file pone.0205348.s012.pdf]

How to use the classification macro:

Download the test datasets and the Fiji macro “Classification” on our homepage:

https://www.biozentrum.uni-wuerzburg.de/bioinfo/computing/3dart-veselect/

- Used Fiji version: 1.51n (download for Unix systems is provided on our homepage)

You can try out the classification macro by using the provided test data from our homepage or you can use your own data sets. Therefore, first of all you need to use 3D ART VeSElecT (download and description on our homepage). The output stacks “StackSegmented” and “StackDuplicateScale” of 3D ART VeSElecT are used as input for the classification macro.

Getting started:

- Open Fiji (prerequisite: 3D ImageJ Suite Plugin is installed)
- Open image test stacks: “StackDuplicateScale.tif” and “StackSegmented.tif”
- Open “Classification” macro

Start classification macro:

- Start the classification by clicking [Run] on the classification macro window (left down corner)

  → A user input window pops up, see Fig1

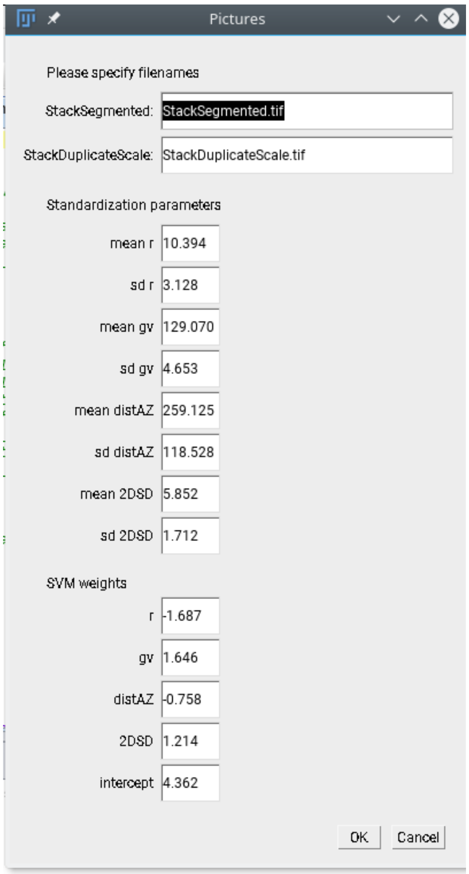

Fig. 1: user input window for names and values: stack names, standardization parameters and SVM weights

Either…

1. **Use default parameters:** If you use the test-data, provided on our homepage, you can skip the following chapter, since you can just use the default parameters. Confirm the parameters by clicking [OK]. Continue reading at: Application of classification macro.

… OR …

2. **Use own data:**

- Type in the name of your segmentation image in line one “StackSegmented”
- Type in the name of your tomogram image in line two “StackDuplicateScale”

➔ Note: If your image stack was saved before, e.g. as a “.tif” data, you need to type in the stack name including the data format ending, e.g. “StackSegmented.tif”

If you use your own electron tomography datasets and want to classify clear cores vesicles from dense core vesicles, try the default parameters first. If results are not

satisfying you probably have to retrain the support vector machine SVM (see retrain SVM tutorial).

➔ If you use your own data and retrained the SVM, please continue reading:

If you use own data and retrained the SVM:

Filenames:

- Type in the name of your stack that includes the segmentation information from 3D ART VeSElecT (default: “StackSegmented.tif”)
- Type in the name of your tomogram stack that includes the scaled (1px/nm) original tomogram information from 3D ART VeSElecT (default: “StackDuplicateScale.tif”)

Standardization parameters:

For standardization of the feature values, the software needs the mean values and standard deviation parameters of each feature of all your data sets. If you apply the classification macro the first time using the default parameters, you will get an output including: radius (r), gray value (gv), distance to the active zone (distAZ), gray value standard deviation (2DSD) for each vesicle. Use this results for calculation of the mean value and standard deviation for each dataset. Afterwards use the results as input in the macro. You can either type the values into the user input window or change the parameters directly at the beginning of the macro script.

SVM weights:

These weight are the output information of the calculated hyperplane of the SVM in the python scikit library. Type in your new weight into the user input window and confirm by clicking [OK]. If you want to use your parameters again without typing them in each time you want to analyze a dataset, change the parameters directly at the beginning of the macro script.

Continuing: Application of classification macro:

- An user interface appears requesting the user to make a point selection right into the center of the active zone of the synapse

  → use the slider to slide through the tomogram images and look for the center of the dense projections

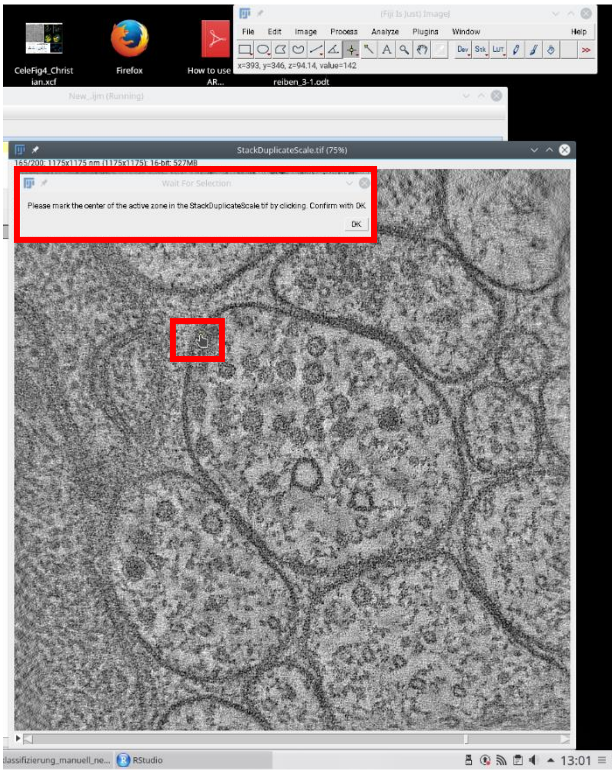

- Make a point selection in the picture “StackDuplicateScale” to mark the center of the active zone
- Confirm with [OK]

Output:

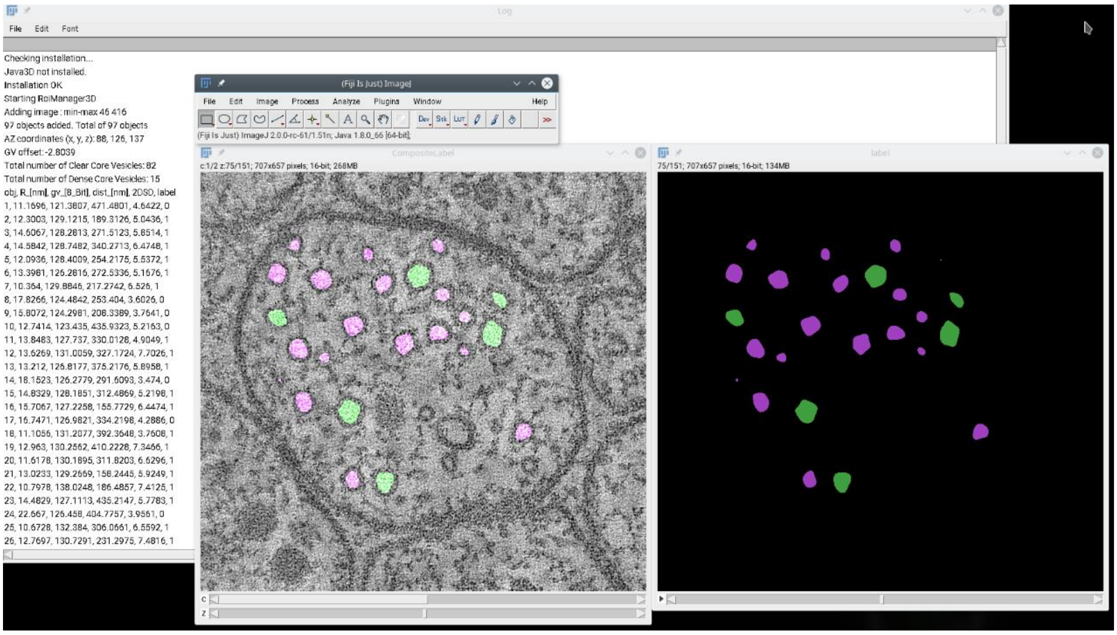

- “Composit” and “StackSegmented” stack with vesicles colored after class (dense core vesicles in green and clear core vesicles in magenta)
- **Results table:**
    - total number of clear core vesicles and dense core vesicles
    - radius
    - gray value
    - distance to the active zone
    - gray value standard deviation

… for each vesicle

    - last column includes labels: 1 = clear core vesicles, 0 = dense core vesicles

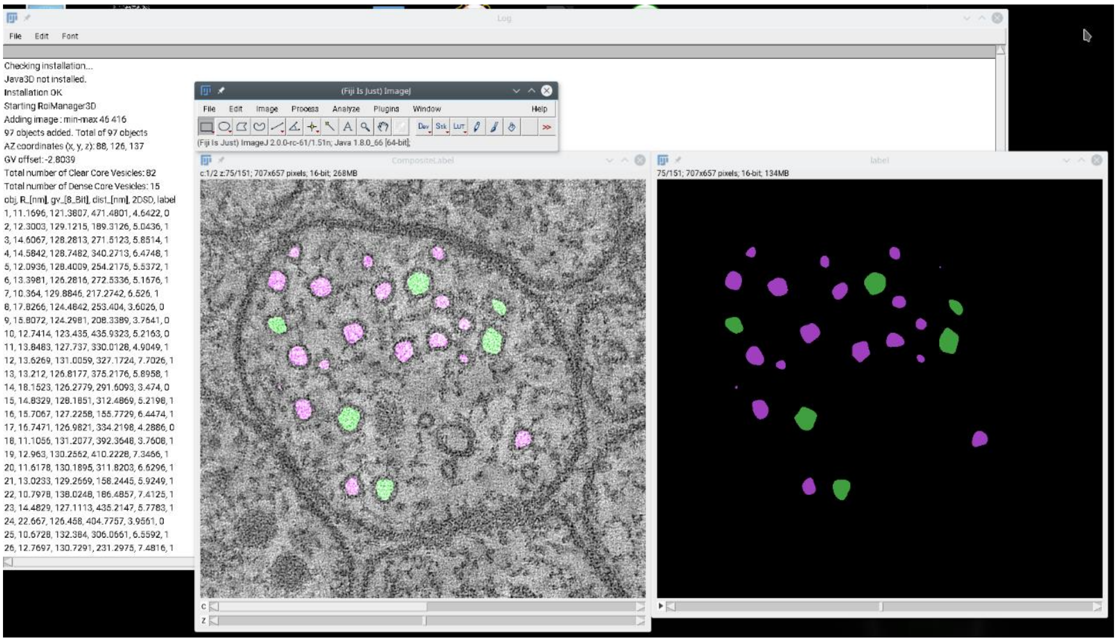
Log
File Edit Font
Checking installation...
Java3D not installed.
Installation OK
Starting RoiManager3D
Adding image : min-max 46 416
97 objects added. Total of 97 objects
AZ coordinates (x, y, z): 88, 126, 137
GV offset: -2.8039
Total number of Clear Core Vesicles: 82
Total number of Dense Core Vesicles: 15
obj, R_[nm], gv_[8_Bit], dist_[nm], 2DSD, label
1, 11.1696, 121.3607, 471.4801, 4.6422, 0
2, 12.3003, 129.1215, 189.3126, 5.0436, 1
3, 14.6067, 128.2813, 271.5123, 5.8514, 1
4, 14.5842, 128.7482, 340.2713, 6.4748, 1
5, 12.0936, 128.4009, 254.2175, 5.5372, 1
6, 13.3981, 126.2816, 272.5336, 5.1676, 1
7, 10.364, 129.8846, 217.2742, 6.526, 1
8, 17.8266, 124.4842, 253.404, 3.6026, 0
9, 15.8072, 124.2981, 208.3389, 3.7641, 0
10, 12.7414, 123.435, 435.9323, 5.2163, 0
11, 13.8483, 127.737, 330.0128, 4.9049, 1
12, 13.6269, 131.0059, 327.1724, 7.7026, 1
13, 13.212, 126.8177, 375.2176, 5.8958, 1
14, 18.1523, 126.2779, 291.6093, 3.474, 0
15, 14.8329, 128.1851, 312.4869, 5.2198, 1
16, 15.7067, 127.2258, 155.7729, 6.4474, 1
17, 16.7471, 126.9821, 334.2198, 4.2886, 0
18, 11.1056, 131.2077, 392.3648, 3.7608, 1
19, 12.963, 130.2562, 410.2228, 7.3466, 1
20, 11.6178, 130.1895, 311.8203, 6.6296, 1
21, 13.0233, 129.2669, 158.2445, 5.9249, 1
22, 10.7978, 138.0248, 186.4857, 7.4125, 1
23, 14.4829, 127.1113, 435.2147, 5.7783, 1
24, 22.667, 126.458, 404.7757, 3.9561, 0
25, 10.6728, 132.384, 306.0661, 6.5592, 1
26, 12.7697, 130.7291, 231.2975, 7.4816, 1
(Fiji Is Just) ImageJ
File Edit Image Process Analyze Plugins Window Help
(Fiji Is Just) ImageJ 2.0.0-rc-61/1.51n; Java 1.8.0_66 [64-bit];
CompositeLabel
c:1/2 z:75/151; 707x657 pixels; 16-bit; 268MB
label
75/151; 707x657 pixels; 16-bit; 134MB
